# Cell-based and cell-free biocatalysis for the production of d-glucaric acid

**DOI:** 10.1186/s13068-020-01847-0

**Published:** 2020-12-10

**Authors:** Lu-Zhou Chen, Si-Ling Huang, Jin Hou, Xue-Ping Guo, Feng-Shan Wang, Ju-Zheng Sheng

**Affiliations:** 1 Key Laboratory of Chemical Biology of Natural Products (Ministry of Education), School of Pharmaceutical Sciences, Cheeloo College of Medicine, Shandong University, Jinan, 250012 China; 2 Bloomage BioTechnology Corp., Ltd., Jinan, 250010 China; 3 The State Key Laboratory of Microbial Technology, Shandong University, Qingdao, 266237 China; 4 National Glycoengineering Research Center, Shandong University, Jinan, 250012 China

**Keywords:** d-Glucaric acid, *Escherichia coli*, Yeast, Metabolic engineering, Cell-free synthetic biology, Biosensor

## Abstract

d-Glucaric acid (GA) is a value-added chemical produced from biomass, and has potential applications as a versatile platform chemical, food additive, metal sequestering agent, and therapeutic agent. Marketed GA is currently produced chemically, but increasing demand is driving the search for eco-friendlier and more efficient production approaches. Cell-based production of GA represents an alternative strategy for GA production. A series of synthetic pathways for GA have been ported into *Escherichia coli*, *Saccharomyces cerevisiae* and *Pichia pastoris*, respectively, and these engineered cells show the ability to synthesize GA de novo. Optimization of the GA metabolic pathways in host cells has leapt forward, and the titer and yield have increased rapidly. Meanwhile, cell-free multi-enzyme catalysis, in which the desired pathway is constructed in vitro from enzymes and cofactors involved in GA biosynthesis, has also realized efficient GA bioconversion. This review presents an overview of studies of the development of cell-based GA production, followed by a brief discussion of potential applications of biosensors that respond to GA in these biosynthesis routes.

## Background

d-Glucaric acid (GA), also called glucarate or saccharic acid, is a naturally occurring aldaric acid in animals and several types of fruits and vegetables [1, 2]. This compound has been used in many fields, including the chemical, food, pharmaceutical, and therapeutic industries [3]. From 2004, GA was classified as a “top value-added chemical from biomass” by the United States Department of Energy because of its potential applications as a material for making biodegradable detergents and biodegradable polymers such as nylons and plastics [4]. As it combines well with metal ions, it is also used as an imaging agent in tumor observation, a surfactant in sewage treatment, and a decolorizer in the treatment of synthetic dyes [5–7]. In medicine, GA is used to reduce cholesterol and suppress tumor development [1]. GA could also enhance human immunity and reduce cancer risks if used as a food additive [8].

**Fig. 1:**
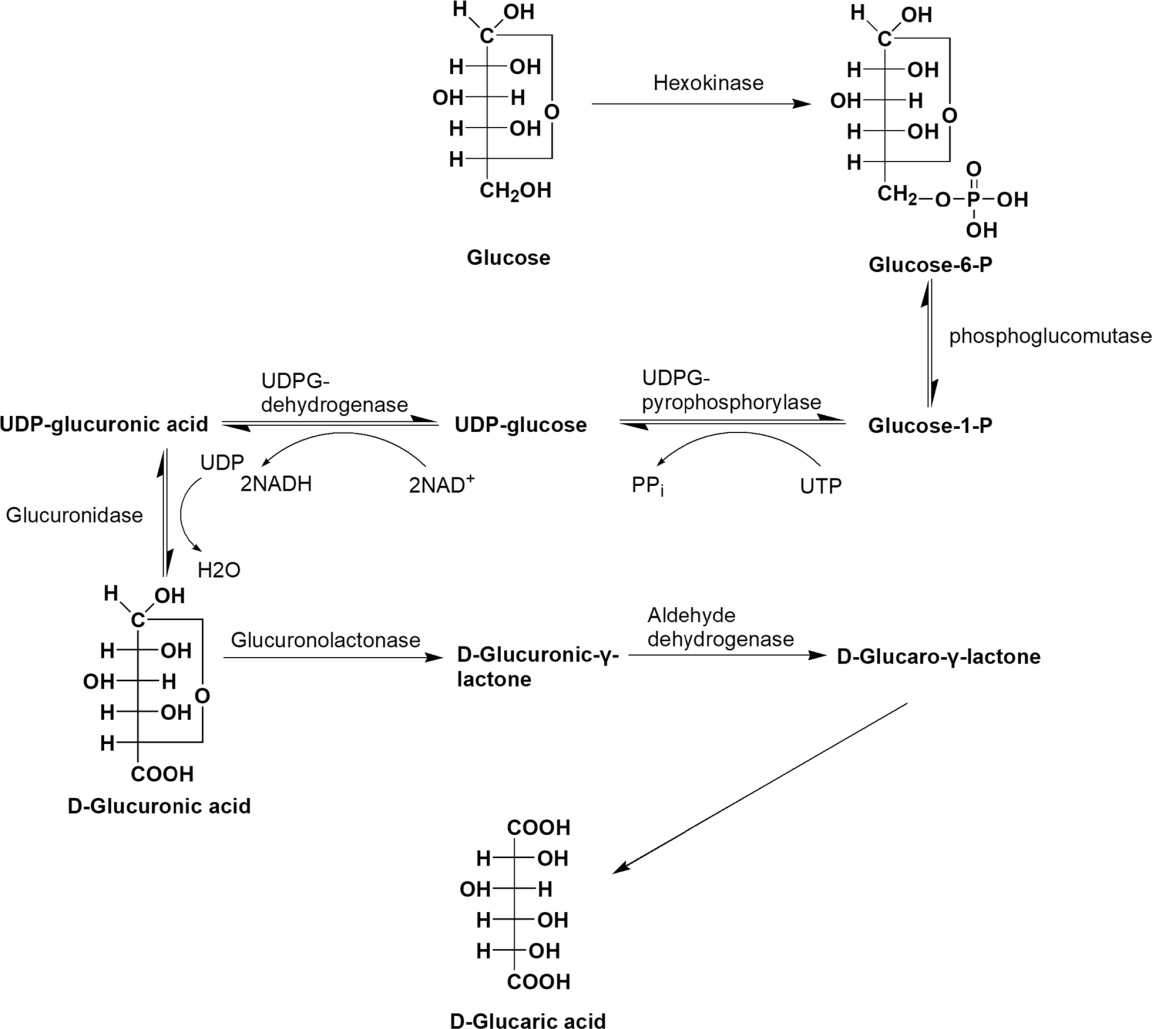
Glucuronic acid (GlcA) pathway and glucaric acid (GA) biosynthesis in natural cells. Glucose-6-phosphate (G6P) formed from glucose is isomerized into glucose-1-phosphate (G1P) by phosphoglucomutase, and then G1P reacts with uridine triphosphate (UTP) [catalyzed by uridine 5′‑diphosphate-glucose (UDP-Glc) pyrophosphorylase] to form UDP-Glc, which is then oxidized in a two-step process by an NAD^+^-dependent UDP-Glc dehydrogenase to form UDP-GlcA. UDP-GlcA is hydrolyzed to form UDP and d-GlcA. The latter then undergoes a three-step conversion reaction through the intermediates d-glucuronic-γ-lactone and d-glucaro-γ-lactone to form GA [9]. However, natural GA is found only at pmol or nmol concentrations in plant and mammalian cells, too low for it to be obtained directly [3]

GA is a by-product of the glucuronic acid metabolism pathway (Fig. 1) in animal and plant cells [9]. This quantitatively minor route of glucose metabolism catalyzes the conversion of glucose to glucuronic acid (GlcA), ascorbic acid, and pentoses, and also provides biosynthetic precursors (nucleotide sugars) to synthesize glycans. In this pathway, glucose-6-phosphate (G6P) formed from glucose is isomerized into glucose-1-phosphate (G1P) by phosphoglucomutase. G1P reacts with uridine triphosphate, catalyzed by uridine 5′-diphosphate-glucose (UDP-Glc) pyrophosphorylase, to form UDP-Glc, which is oxidized in a two-step process by an NAD^+^-dependent UDP-Glc dehydrogenase to UDP-GlcA. UDP-GlcA is hydrolyzed to form UDP and d-GlcA. Then the latter undergoes a three-step conversion reaction through the intermediates d-glucuronic-γ-lactone and d-glucaro-γ-lactone to form GA. However, GA is found in plant and mammalian cells at only pmol or nmol concentrations, which is too low for it to be obtained directly [3].

Thus, marketed GA is currently produced chemically. First, a nonselective oxidation reaction using nitric acid was developed [10]. Though optimized, the molar ratio of nitric acid to glucose was 4:1, causing great damage to the environment and had a low conversion from glucose to GA (< 50% yield) [11]. This situation was not changed until the 1960s when the compound 2,2,6,6-tetramethyl-1-piperidinyloxy (TEMPO) was discovered [12]. This material functions as a catalyst in the oxidation of alcohols to aldehydes. It means the reaction can be performed in milder conditions, with production of more of the target compound (70–90% yield) [13–16]. However, because the chemical oxidation method for GA production from glucose is not selective, it is difficult to obtain a single compound, and the production of nitrogen compound byproducts is environmentally unfriendly [11]. Therefore, it is highly desirable to develop environmentally-friendly and cost-effective biosynthesis strategies to produce GA. Metabolic engineering and in vitro multiple-enzyme catalysis are effective strategies, and have leapt forward in recent years [17]. Indeed, metabolic engineering and in vitro multiple-enzyme catalysis are effective strategies, and have leapt forward in recent years [17]. This review provides an introduction to synthetic-biology-based methodologies for high-yield GA-biosynthesizing cells (*Escherichia coli* and yeast) (Table 1), followed by a discussion of in vitro multiple-enzyme catalysis approaches to produce GA.

**Table 1:** Summary of bio-based production of d-glucaric acid. Modified from Ref. [69]

	Year	Hosts	Genes	Carbon sources	Titer (g/L)	Yield (g/g)	Refs.
In vivo	2009	*E. coli* BL21 Star (DE3)	*ino1*, *MIOX*, *udh*	Glucose	1.13	0.113	[18]
	2010	*E. coli* BL21 Star(DE3)	*ino1*, *MIOX*, *udh*	Glucose	2.50	0.250	[22]
	2014	*E. coli* MG1655	*ino1*, *SUMO-MIOX*, *udh*	Myo-inositol	4.85	0.449	[26]
	2015	*E. coli* IB1486	*ino1*, *SUMO-MIOX*, *udh*	Glucose	1.56	0.124	[44]
	2017	*E. coli* L19S	*ino1*, *MIOX*, *udh*	Glucose	> 0.8	–	[45]
	2018	*E. coli* K-12	*ino1*, *MIOX*, *udh*	Glucose	1.98	0.198	[48]
	2018	*E. coli* BL21 Star(DE3)	*cscB*, *cscA*, *cscK*, *ino1*, *MIOX*, *udh*, *suhB*	Sucrose	1.42	0.27	[19]
	2020	*E. coli* BL21 Star(DE3)	*ino1*, *MIOX*, *udh*, *suhB*	Glucose	5.35	0.467	[40]
	2016	*S. cerevisiae* CEN.PK2-1D *opi1Δ*	*ino1*, *inm*, *MIOX*, *udh*	Glucose	0.98	0.33	[50]
	2016	*S. cerevisiae* CEN.PK2-1D *opi1Δ*	*ino1*, *inm*, *MIOX*, *udh*	Glucose, myo-inositol	1.6	–	[50]
	2016	*P. pastoris* GS115	*MIOX*, *udh*	Glucose, myo-inositol	6.61	–	[50]
	2018	*S. cerevisiae* BY4741 *opi1∆*	*ino1*, *MIOX*, *udh*	Glucose, myo-inositol	6.0	–	[55]
	2020	*S. cerevisiae*	*ino1*, *inm*, *MIOX*, *udh*, *vhB*	Glucose	6.38	0.128	[58]
In vitro	2016	Soluble enzymes	*xyn*, *AG*, *udh*	Hemicellulose	–	–	[67]
	2019	Soluble enzymes	*SP*, *PGM1*, *MIPS*, *IMPASE*, *MIOX*, *udh*, *nox*	Sucrose	7.31	0.75	[64]
	2020	Soluble enzymes	*AxyAgu115A*, *GOOX*	Xylan	–	–	[68]
	2020	Immobilized enzymes	*PGM1*, *IPS*, *IMP*, *MIOX*, *udh*, *nox*	Glucose-1-phosphate	1.70 ± 0.04	0.202	[69]

## Construction and optimization of heterologous GA synthesis pathways in *E. coli*

### Construction of heterologous GA synthesis pathways in *E. coli*

**Fig. 2:**
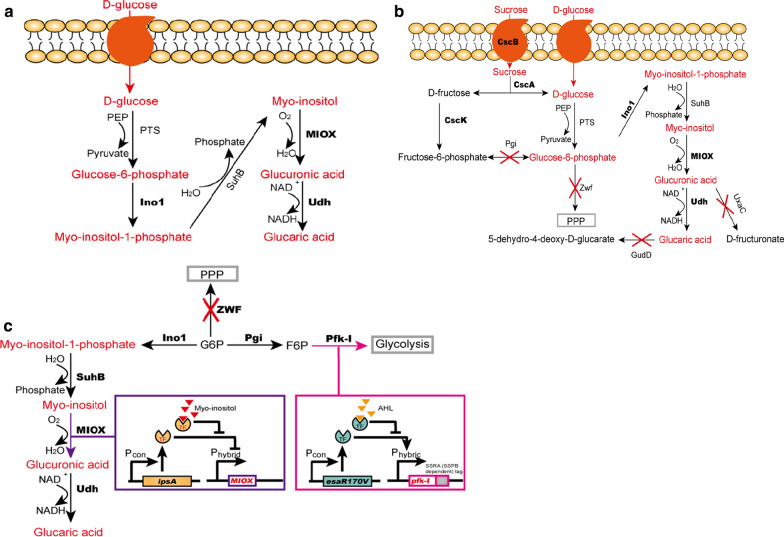
**a** Schematic overview of heterologous d-glucaric acid (GA) synthesis pathways in engineered *Escherichia coli* starting from glucose [18]. Red text indicates key substances in the synthesis pathway of GA. Black and bold text represent heterologous enzymes involved in the production of GA. **b** Sucrose is used as an alternative substrate for GA synthesis in vivo and some genes are knocked out to increase GA production. Some genes related to the consumption of GA synthesis intermediates, such as G6P, GlcA, and GA, have been knocked out. A red cross indicates that enzymes in the relevant metabolic pathway have been eliminated [25]. **c** Dynamic regulation of genes to increase GA production. The means of sending G6P to glycolysis in pink arrow is to dynamically knock-down Pfk-I, whose expression is controlled by a quorum-sensing system [45]. A degradation tag is fused with Pfk-I and it rapidly loses activity in the presence of SspB. The black “TF” indicates transcriptional activator protein EsaR170V. In the pathway of conversion of G6P to GA, the expression of MIOX has been dynamically controlled by a biosensor-based promoter regulated by the MI-specific sensor IpsA. The red “TF” indicates transcriptional repressor IpsA. *CscA* invertase, *CscB* sucrose permease, *CscK* d-fructokinase, *GudD* glucarate dehydratase, *Ino1* myo-inositol-1-phosphate synthase, *MIOX* myo-inositol oxygenase, *PGI* phosphoglucose isomerase, *Pfk-I* phosphofructokinase-1, *PPP* pentose phosphate pathway, *PTS* phosphotransferase, *SuhB* inositol-1-monophosphatase, *Udh* uronate dehydrogenase, *UxaC* uronate isomerase, *Zwf* glucose-6-phosphate dehydrogenase

The first successful example of GA biosynthesis in microbes using a metabolic engineering approach was the manipulation of recombinant *E. coli* to produce GA (Fig. 2a). This Gram-negative bacterium is a very versatile host for the production of heterologous proteins, such as those within the natural GA synthesis pathway. In 2009, Moon et al. successfully constructed a recombinant *E. coli* strain producing GA from glucose by heterologously co-expressing three enzymes: myo-inositol-1-phosphate synthase (Ino1) from *Saccharomyces cerevisiae*, which converts G6P into myo-inositol-1-phosphate (I1P); myo-inositol oxygenase (MIOX) from mouse, which converts myo-inositol (MI) into GlcA with the consumption of one molecule of oxygen; and *Pseudomonas syringae* uronate dehydrogenase (Udh), which converts the GlcA into GA using NAD^+^ as a cofactor. The final titer of GA from glucose using this strain reached 1.13 g/L and the yield was 0.113 g/g glucose [18]. However, it was found that MIOX is intrinsically unstable, which directly limited the process of MI conversion to GlcA [18]. Furthermore, a considerable fraction of the carbon source (such as glucose in M9 medium) is used for cell growth and only a small fraction enters the artificially constructed GA production pathway [18], which demonstrates that the competition of various metabolic pathways in cells is also one of the factors that affects GA production.

Meanwhile, some researchers have tried to use other sugars as substrates for synthesis of GA. Sucrose was used as the substrate rather than d-glucose [19]. Sucrose has the advantages of being low cost and easy to obtain [20]. Compared with the pathway starting from d-glucose, the pathway starting from sucrose requires three additional enzymes, invertase (CscA), sucrose permease (CscB) and d-fructokinase (CscK) from *E. coli* strain W (Fig. 2b). These enzymes could make *E. coli* have the ability of consuming sucrose and convert sucrose into d-glucose and d-fructose; the former monosaccharide is then used for GA synthesis, while the d-fructose acts as a carbon source that improves the cell growth compared with use of d-glucose as the sole carbon source.

### Synthetic scaffolds for multiple enzymes in *E. coli*

Co-expression of multiple enzymes on a scaffold in one cell is an effective strategy to increase GA synthesis efficiency. Use of synthetic scaffolds in GA-synthesizing *E. coli* strains allows greater channeling of intermediates through improved flux, stabilization of intermediates, and protection from other reactions [21]. A scaffold carrying specific ligands combining the functional domains of Ino1 and MIOX was constructed in vivo, which achieved enzyme colocalization and greatly improved the activity of MIOX. The production of GA starting from glucose reached 2.5 g/L, 5 times higher than when the enzymes without the scaffold were expressed from the same vectors and the yield of GA was 0.25 g/g glucose [22].

### Optimization of metabolic routes through elimination of competing enzymes

The knockout of competing genes has been used to improve the conversion ratio of strains for some chemical products [23]. G6P is a primary material for cell growth, and takes part in the Enter–Dudoroff and Embden–Meyerhoff–Parnas pathways in the cell [24]. After introducing the GA production pathway into *E. coli*, it was found that a large part of G6P entered the glycolytic pathways and was used for cell growth at the initial stage (Fig. 2b). Considering it is an intermediate in the heterologous synthesis of GA, genes encoding glucose-6-phosphate dehydrogenase (*zwf*) and phosphoglucose isomerase (*pgi*) were knocked out from the *E. coli* chromosome, resulting in more G6P accumulation in the cell and either glycerol, xylose, or arabinose was used as carbon sources instead of glucose for cell growth [25]. Furthermore, genes encoding uronate isomerase (*uxaC*) and glucarate dehydratase (*gudD*) were knocked out to increase the amount of the intermediate d-GlcA and decrease the consumption of the product GA [26].

### Optimization of the rate-limiting enzyme—MIOX

It has been become generally accepted that MIOX is the rate-limiting enzyme within the artificial GA biosynthesis pathway in *E. coli*. Natural MIOX is part of a pathway for the catabolism of inositol in kidneys [2]. It was first identified in 1957 from extracts of rat kidney [27]. However, its catalytic mechanism was not determined until the 1980s because of its poor stability in vitro: this non-heme di-iron enzyme employs a unique four-electron transfer at its Fe(II)/Fe(III) coordination sites, and the reaction proceeds through the attack of the iron center by diatomic oxygen followed by abstraction of the MI hydrogen atom [28]. Therefore, the reaction catalyzed by MIOX needs Fe^2+^ and cysteine both in vitro (for purified recombinant MIOX) and in recombinant *E. coli* [29]. Strategies to make this artificial GA biosynthesis pathway more effective include improving the expression level of soluble MIOX in recombinant cells and using mutants with better catalytic activity instead of wild-type MIOX. In *E. coli*, mouse MIOX, a protein from eukaryotic cells, possibly fails to fold properly, instead forming aggregates and precipitating as inclusion bodies, resulting in a limited level of soluble MIOX. A small ubiquitin-related modifier (SUMO)-tag, a commonly used fusion tag to enhance expression and solubility of recombinant proteins in *E. coli* [30], was fused at the N-terminus of MIOX and the yield of GA increased 125% compared with the use of wild-type MIOX [26].

Directed evolution has emerged in the past few years as a powerful tool that is widely used to modify the properties of enzymes [31–34]. Usually, in the absence of sufficient information about the protein structure and catalytic mechanism, common mutation methods such as error-prone PCR and chemical mutagens are applied to obtain a large number of mutants of the desired protein [35]. The key to the success of directed evolution is a suitable high-throughput screening method depending on selective protein properties that can be used to screen the desired protein efficiently [36]. There are two methods to screen MIOX. One is to use the native metabolism of *E. coli* to couple the activity of MIOX with the growth rate of the strain [26]. *E. coli* cannot grow when MI is used as the sole carbon source. However, when MIOX converts MI into GlcA, *E. coli* can consume GlcA as the carbon source and grow. Therefore, the screening of high-activity MIOX mutants can be achieved by screening the cellular growth rate when using MI as the sole carbon source. Although the authors did not identify a high-activity MIOX mutant using this screening method, they unexpectedly discovered a DNA fragment that could increase MI transport [26]. In this better GA producing host, the wild-type *MIOX* gene was mutated and a 941-bp DNA insertion fragment occurred downstream of the MIOX terminator. GA production was obviously decreased when the 941-bp DNA fragment was removed. The authors analyzed the DNA fragment and found that the proteins encoded by this DNA fragment could enhance the transportation of MI into the cell [26]. Another method of screening for desired MIOX mutants is using a GA-responsive biosensor [37]. Based on synthetic tools such as biosensors, high-throughput screening strategies using individual recombinant *E. coli* cells as a sorting section are playing growing roles in the directed evolution of enzymes involved in GA biosynthesis. A biosensor that responds to GA has been designed and successfully used in identification of desired MIOX orthologs and GA-producing strains [37, 38]. Recently, several highly active mutants of MIOX have been identified through an effective high-throughput screening approach (a one-pot two-strain system) based on a GA biosensor system [37]. D82Y and S173N mutants of MIOX displayed approximately 3.8- and 2.7-times higher than the wild-type activity toward MI in a broad pH range (pH 3–6).

Ribosome binding site (RBS) optimization is another strategy to enhance GA production by coordinating the expression of enzymes [39]. The activity ratio of Ino1 to MIOX was improved from 1:0.40 to 1:3.41, which greatly enhanced the GA titer starting from glucose, to 4.56 g/L, compared with the unoptimized strain (3.42 g/L) and the yield of GA was 0.467 g/g glucose [40].

### Dynamic control of GA synthetic pathways

Dynamic control is an important tool in metabolic engineering that allows global optimization of flux of carbon in vivo. Phosphofructokinase (Pfk-I) is a critical enzyme controlling the flux of G6P into glycolysis that influences cell growth. Meanwhile, G6P is an important intermediate in GA synthetic pathways, and in vivo self-regulation of Pfk-I expression, G6P could be switched between glycolysis and GA metabolic pathway (Fig. 2c). Additionally, it would maximize GA yields and minimize the need for manual intervention in the fermentation processes. Firstly, 10Sa RNA (*ssrA*) was added to the coding sequence of *pfk-I* to enable dynamic degradation of Pfk-I in the cell [41]. This degradation tag would result in the slow degradation of Pfk-I in the absence of stringent starvation protein B (SspB) and rapid degradation in the presence of SspB which tethers target proteins to the protease ClpXP, resulting in the accumulation of G6P, the precursor of MI, in the cells [42, 43]. Based on this, a relationship between the activity of Pfk-I and the expression of SspB was established: the more SspB was expressed, the less Pfk-I was present in the cell. The expression of SspB was controlled by an inducible promoter. In this way, the strain of overexpressing SspB redirected more G6P to the GA intermediate MI by controlling the degradation of Pfk-I. As expected, *E. coli* carrying this gene showed the 1.56 g/L of the GA titer from glucose, a 42% improvement over the strain without *sspB* and the yield of GA was 0.124 g/g glucose [44].

In a development of this approach, a modified and self-constructed quorum sensing (QS) system was developed to achieve dynamic expression of Pfk-I protein [45]. QS is a common mechanism by which bacteria regulate gene expression based on cell density using signal molecules [46]. The synthesis of capsular polysaccharide (CPS) is a typical process controlled by QS, and the histone acetyltransferase Esa1 that acts as a transcriptional activator of CPS synthesis is induced by the level of 3-oxohexanoyl-homoserine lactone (AHL) [47]. As mentioned before, Pfk-I takes part in many important reactions in the process of cell growth, and knocking out Pfk-I causes the accumulation of G6P. Thus, by controlling the expression of Pfk-I, “cell growth” and “GA production” modes could be achieved (Fig. 2c). Initially, the cells would be in “cell growth” mode when Pfk-I was expressed in the cells under the control of the promoter of *esaS* (P_esaS_) in the presence of regulation by the protein activator EsaR170V. Then, when the cell density reached a desired value, expression of Pfk-I would stop as the AHL interacted with the EsaR170V, resulting in low transcription of the *pfk-i* gene from P_esaS_. A standard SSRA degradation tag was appended to the C-terminus of Pfk-I altering the half-life of which to ensure the Pfk-I protein could be removed rapidly when the transcription halted. Using the QS system to control the expression of Pfk-I, the “cell growth” mode would be switched to the “GA production” mode when the cell population reached a desired level. In this engineered system, the final MI titer increased 5.5-fold and the GA titer fourfold higher compared with the strain with *pfk-i* controlled by its native promoter [45].

Lastly, the dynamic control method was also used to control the expression of MIOX, to avoid loss of catalytic activity over time in *E. coli* (Fig. 2c)*.* Briefly, a hybrid promoter that could sense the concentration of MI to control the expression of MIOX was used [48]. In the absence of MI, the transcription factor IpsA could bind with this promoter and recruit RNA polymerase to activate *MIOX* gene expression [49]. In the presence of MI, MI binds to IpsA, causing the IpsA to dissociate from DNA, hence decreasing *MIOX* gene expression. Then, based on this system, a hybrid promoter that could sense MI with the opposite mechanism was engineered: expression of the gene of interest was repressed by blocking access to RNA polymerase in the absence of MI. Binding of MI to IpsA would cause structural changes of IpsA which would dissociate from the promoter, activating expression of the gene of interest. Using the MI-responsive promoter to control MIOX expression, the GA titer from glucose was increased 2.5-fold compared with the unregulated MIOX control. Finally, when combining the dynamic control method to regulate the expression of Pfk-I and MIOX, the final GA titer was 1.98 g/L, nearly fivefold increase compared with that when only Pfk-I was regulated (0.4 g/L) and the yield of GA was 0.198 g/g glucose [48].

## Construction and optimization of heterologous GA synthesis pathways in yeast

**Fig. 3:**
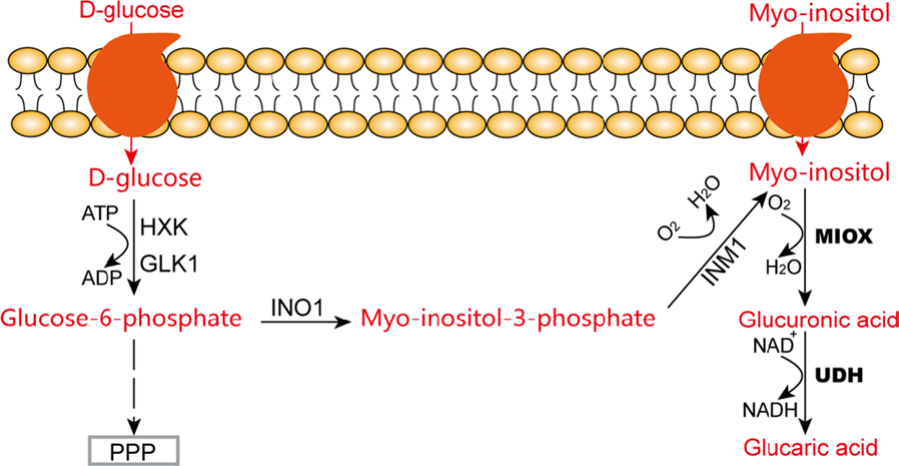
Schematic overview of heterologous GA synthesis pathways in engineered yeast [50, 55, 58]. Red text indicates key substances in the synthesis pathway of GA. Black and bold text represent heterologous enzymes involved in the production of GA. Myo-inositol (MI) or glucose can be used as the substrate for GA synthesis in vivo. *GLK1* glucokinase-1, *HXK* hexokinase, *Ino1* myo-inositol-1-phosphate synthase, *Inm1* inositol monophosphatase 1, *MIOX* myo-inositol oxygenase, *PPP* pentose phosphate pathway, *Udh* uronate dehydrogenase

Engineering *E. coli* cells for GA production requires mitigation of the limitations that arise from the inherent toxicity of GA toward *E. coli* [30]. Yeast cells are more resistant to toxicity resulting from a low pH environment than *E. coli*. Thus, the GA biosynthesis pathway has been introduced into yeasts such as *S. cerevisiae* and *Pichia pastoris* [50]. The former species has “generally recognized as safe” status, and is widely used in the metabolic engineering field for production of industrial materials such as bioethanol, and bulk and fine chemicals [51, 52]. *P. pastoris* is increasing in use as an alternative host to *S. cerevisiae* because it can be fermented to very high density, enabling high level production of target compounds [53]. Unlike *E. coli*, yeasts have native forms of Ino1 and inositol monophosphatase (Inm1) that convert G6P to MI (Fig. 3). Therefore, introducing the heterologous enzymes MIOX and Udh could give cells GA producing ability. However, conversion from MI to GlcA catalyzed by MIOX is less active than the other enzyme-catalyzed steps in the artificial GA biosynthesis pathway in recombinant yeast [50]. To explore candidate genes encoding MIOX that were better suited for heterologous overexpression in yeast, *MIOX* genes from *Arabidopsis thaliana* were employed to replace the role of *MIOX* from *Mus musculus*, and introduced into the GA biosynthesis pathway [50]. Compared with MIOX from *Mus musculus*, higher GA production, ~ 0.5 g/L, was observed when the recombinant *S. cerevisiae* carried the *A. thaliana miox* gene. Chen et al*.* tried to solve the problem of low catalytic efficiency of MIOX by integrating the *MIOX* gene from *A. thaliana* and *udh* from *P. syringae* into a delta (δ)-sequence. The δ-sequence is usually employed to increase the expression of protein under the control of strong promoters, such as the promoter of glyceraldehyde-3-phosphate dehydrogenase [54]. The engineered strain produced 3.8 g/L GA from myo-inositol in shake flask culture, 7.04 times higher than that of the strain without integration of genes into the δ-sequence. The final GA starting from glucose and myo-inositol reached 6 g/L after the fed-batch fermentation [55]. In 2020, to identify better candidates for GA production in *S. cerevisiae*, proteins in the UniProt database with sequence similarity to MIOX family members together with Udh were, respectively, introduced into recombinant yeast cells harboring the pathway form glucose to GA [56]. In this study, 25 novel homologues were characterized, and displayed the ability to convert MI to GA together with Udh. It is worth mentioning that the recombinant cells expressing *Talaromyces marneffei* MIOX produced more GA, 1.76 ± 0.33 g/L GA from 20 g glucose/L and 10 g/L MI, than cells expressing the often-used *Arabidopsis thaliana* variant AtMiox4.

In a recent report, it was found that titers of GA could be improved by introducing *Vitreoscilla* hemoglobin into cells to increase the amount of dissolved oxygen [57]. In the conversion of MI to GlcA, MIOX acts as a monooxygenase and oxygen is needed in the reaction process. The increase of cellular oxygen might increase oxygen availability for this reaction and/or accelerate cell metabolism and/or growth and thus cause more efficient GA production [58]. It is worth mentioning that when *P. pastoris* was employed as the host, recombinant strains produced GA from glucose and MI up to 6.61 ± 0.30 g/L, which is also better than the titer achieved in recombinant *E. coli* [40].

The production of GA correlated with MI availability in recombinant yeast [50]. The final production increased when the medium was supplemented with MI, which indicated that insufficient MI was being synthesized, and this created a bottleneck in GA synthesis in yeast. Unlike in *E. coli*, in yeasts, MI is an important material (produced by endogenous Ino1 and Inm). In this process, the expression of Ino1 is controlled by the negative regulation of OPI1. The expression of Ino1 was greatly increased when the gene *opi1* was knocked out, resulting in a substantial increase in production of the GA precursor MI [59].

## Cell-free multi-enzyme catalysis approaches to produce GA

In vitro multi-enzyme catalysis is another candidate route for biosynthesis of GA. Cell-free multi-enzyme catalysis usually involves construction of a single pathway with several enzymes and coenzymes in vitro. Cell-free multi-enzyme catalysis approaches for GA production are commonly considered to be efficient and environmentally-friendly, and to be a practical solution to the problems posed by fermentation [60]. These issues, such as low conversion rate and unbalanced pathway fluxes, have hampered industrial-scale production of GA using metabolically engineered *E. coli* or yeast strains. Meanwhile, cell-free multi-enzyme catalysis systems have advantages such as high reaction efficiency, easy control of reaction conditions and product separation, and a small environmental footprint [61–63].

**Fig. 4:**
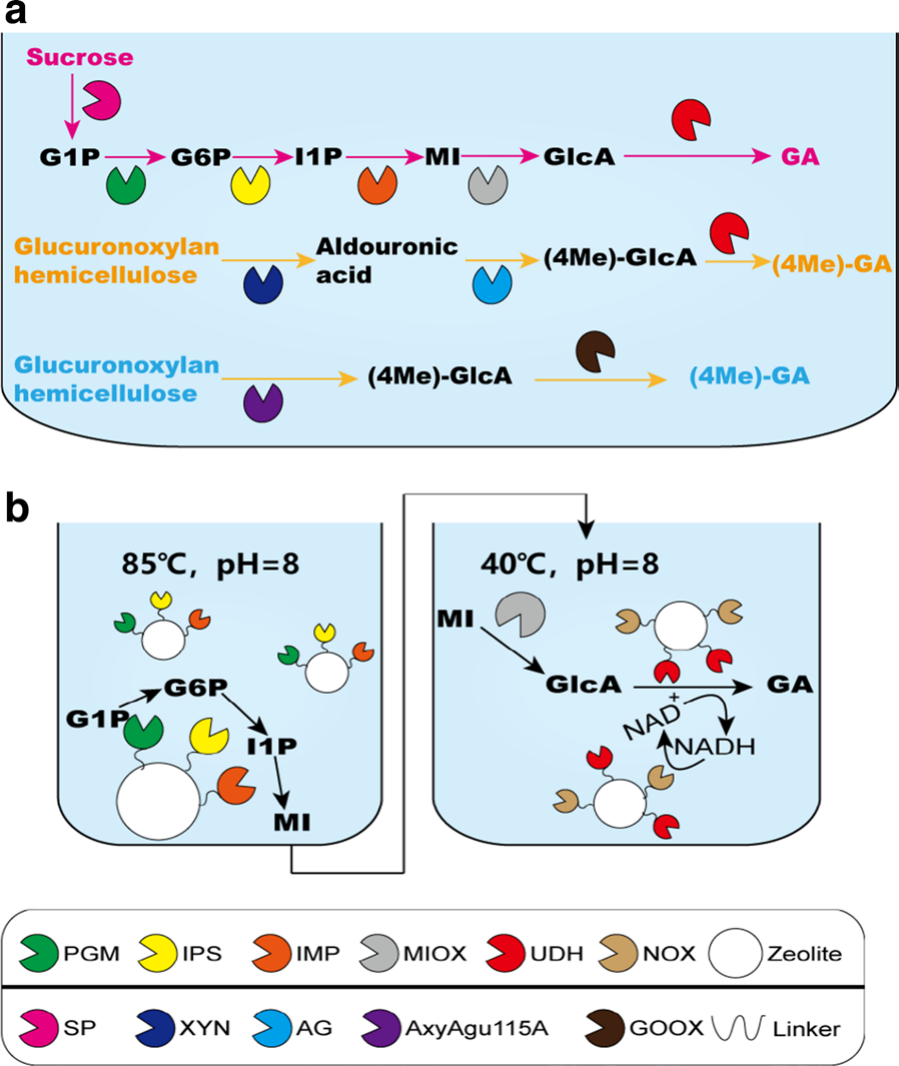
In vitro cascade for production of GA. Four different methods of synthesizing GA in vitro are represented by different colored text and arrows [64, 67–69]. **a** Pink text indicates the GA synthesis route starting from sucrose. Sucrose is converted to G1P by SP, and then GA is produced by the action of five enzymes—PGM, IPS, IMP, MIOX, and Udh. Orange text represents the in vitro synthesis route of GA starting from glucurononxylan hemicellulose. Blue text represents a novel synthesis route of GA starting from glucurononxylan hemicellulose. **b** Black text represents the in vitro synthesis route of GA starting from G1P. The reaction was divided into two parts: high-temperature and low-temperature systems. The enzymes related to the GA pathway were connected to zeolite via a peptide linker. *AG* glucuronidase, *AxyAgu115A* GH115 α-glucuronidase, *G1P* glucose-1-phosphate, *G6P* glucose-6-phosphate, *GOOX* gluco-oligosaccharide oxidase, *I1P* myo-inositol-1-phosphate, *IMP* inositol-1-monophosphatase, *IPS* myo-inositol-3-phosphate, *MI* myo-inositol, *MIOX* myo-inositol oxygenase, *Nox* NADH oxidase, *PGM* phosphoglucomutase, *SP* sucrose phosphorylase, *Udh* uronate dehydrogenase, *XYN* xylanase

In 2019, an in vitro GA biosynthesis method using sucrose as substrate and seven enzymes was established (Fig. 4a) [64]. Briefly, sucrose phosphorylase (SP) converted sucrose into G1P and d-fructose. Then, G1P was transformed into G6P by phosphoglucomutase (PGM). Next, Ino1 converted G6P into I1P. The I1P was dephosphorylated into MI, and finally oxidized to GA by MIOX and Udh. NADH oxidase (Nox) could achieve the recycling of NADH during the GA synthesis process. By optimizing the reaction conditions and enzyme composition, the GA yield was up to 0.7 g/g sucrose from 17 g/L sucrose, and the final concentration of GA was 7.3 g/L. As Nox could regenerate NAD^+^, the reaction only needed 2 g NAD^+^/L. This saved costs and enhanced efficiency. Hemicellulose, the second most abundant form of biomass in plant cell walls after cellulose, is an alternative material that can be employed for efficient conversion into GA [65, 66]. A successful biosynthesis pathway from hemicellulose to GA was constructed (Fig. 4a). The pathway was different from the previously reported pathway constructed in recombinant *E. coli*, and used three enzymes: xylanase (Xyn) from *Flavobacterium* sp., glcuronidase (AG) from a rumen metagenomic library, and Udh from *P. mendocina* [67]. These three enzymes were co-localized into a scaffold and the final product GA increased by 20% compared with the case when the three enzymes were free in solution. However, continuous supplementation with the expensive cofactor NAD^+^ was required in this reaction system, and the soluble xylo-oligosaccharides affect the separation of GA from the reaction system. To avoid these limitations, a novel method of producing GA from hardwood xylan was constructed, in which no supplementation of NAD^+^ was required and the xylan co-product could be easily separated by centrifugation [68]. Briefly, two novel enzymes were introduced into the one-pot reaction (Fig. 4a). A GH115 α-glucuronidase (a member of glycoside hydrolase family 115) from *Amphibacillus xylanus* releases almost all the 4-*O*-methyl d-GlcA from glucuronoxylan. Then, a Y300A mutant of *Sarocladium strictum* gluco-oligosaccharide oxidase converts the glucuronoxylan to 4-*O-*methyl d-GlcA. The yield of GA in this process was 0.62 g/g 4-*O*-methyl d-GlcA [68].

Petroll et al. [69] constructed an immobilized multi-enzyme biosynthesis system, and they successfully overcame the limitations of cell-free catalysis based on immobilization of thermostable enzymes and cofactor regeneration (Fig. 4b). The in vitro GA titer from G1P was 3.0 ± 0.2 g/L for free enzymes, and 1.70 ± 0.04 g/L for immobilized enzymes. Briefly, they divided the GA synthesis reaction into two parts depending on the temperature stability of the enzymes. The thermostable enzymes consisted of phosphoglucomutase (PGM-L), myo-inositol-3-phosphate synthase (IPS-L), and inositol-1-monophosphatase (IMP-L), and using these enzymes, G1P was produced from MI at 85 °C. The non-thermostable enzymes, MIOX, Udh, and Nox, converted MI to GA at 40 °C. In addition, a linker peptide was fused at either the N- or C-terminus of these enzymes so they had good affinity for the silica-based matrix [70].

## High-throughput screening approach based on GA biosensor

It is highly desirable to develop an effective GA concentration screening approach. Promoters responding to GA, and promoters that allow for real-time control of upstream genes, would be an effective solution. Such promoters and their transcriptional regulators would have high value, and enable optimum GA pathway flux and construction of GA biosensors. The biosensors would be powerful during the identification of critical in vivo points for improvement of GA titer, and for directed evolution of enzymes involved in GA biosynthesis. In 2016, Rogers et al. [71] developed a GA biosensor based on the transcription factor CdaR and a 521-bp promoter in *E. coli*. Two years later, an effective high-throughput screening approach was established, and applied in the identification of highly active mutants of MIOX [37]. An *E. coli* strain containing the GA biosensor system could act as a GA “detector”, and with its help, the GA concentration within medium or a reaction mixture could be ranked. Though this approach is not a quantitative analysis method, it is suitable for high-throughput sorting, and a feasible solution to the lack of a fast and effective GA detection method. Yeasts are candidate host cells that may be better suited for the production of GA than *E. coli*, because of their higher resistance to acid toxicity from GA accumulation in vivo. Unfortunately, GA-inducible transcriptional regulators of natural eukaryotic promoters have not been reported, though it is believed that these components would be straightforward and powerful tools for yeast metabolic engineering. Transplantation of prokaryotic transcriptional activators into the eukaryotic chassis is a potential solution to this problem [72–74].

## Conclusion

GA is commonly identified as one of the “top value-added chemicals from biomass”, and is currently produced by chemical method. The alternative is bio-based production approaches. Therefore, the development of GA production based on fermentation and metabolically engineered cells or in vitro cell-free biocatalysis has leapt forward in recent years, and GA titers and yield have increased rapidly [18, 19, 26, 40, 51, 64, 69]. Firstly, a series of synthetic pathways for GA have been ported into *E. coli*, *S. cerevisiae*, and *P. pastoris*, and these engineered cells show the ability to synthesize GA de novo. After abundant works to improve the GA metabolic pathways in host cells, GA titers from glucose have reached around 5 g/L (from glucose) and 6 g/L (from glucose and MI) when *E. coli* and *P. pastoris* are used as host cells, respectively.

Probing protein sequence–function relationships of MIOXs then engineering members of this enzyme family would yield MIOX variants with desired enzymatic activity and increased stability, which would be a fundamentally important step in advancing biobased GA production. Furthermore, tools in synthetic biology are changing rapidly, which will be important for improvement of the efficiency of GA biobased production. There has been recent interest in the dynamic regulation of flux through metabolic pathways to overcome some of the issues arising from the introduction of the GA biosynthesis pathway into host cells. In the future, with a range of metabolite sensors responding to GA directly or to intermediate metabolites within the GA biosynthesis pathway being discovered or artificially constructed, control can be implemented at different dynamic timescales.

Briefly, we expect that continuing advances in synthetic biology and protein engineering will allow the design of efficient and environmentally-friendly approaches to produce GA, and meet the requirements of GA application in medicine and industry.

## Data Availability

No new data generated in this review.

## Abbreviations

AHL: 3-Oxohexanoyl-homoserine lactone
CPS: Capsular poly-saccharide
CscA: Invertase
CscB: Sucrose permease
CscK: d-Fructokinase
EsaR: Acyl-homoserine-lactone synthase repressor
GA: Glucaric acid
GlcA: Glucuronic acid
GudD: Glucarate dehydratase
G1P: Glucose 1-phosphate
G6P: Glucose-6-phosphate
IMP: Inositol-1-monophosphatas
Inm1: Inositol monophosphatase
Ino1: Myo-inositol-1-phosphate synthase
I1P: Myo-inositol-1-phosphate
MI: Myo-inositol
MIOX: Myo-inositol oxygenase
MIPS: Myo-inositol 1-phosphate synthase
Nox: NADH oxidase
Pgi: Phosphoglucose isomerase
PGM: Phosphoglucomutase
Pfk: Phosphofructokinase
QS: Quorum sensing
SP: Sucrose phosphorylase
RBS: Ribosome binding site
SsrA: 10Sa RNA
SspB: Stringent starvation protein B
SUMO: Small ubiquitin-related modifier
TEMPO: 2,2,6,6-Tetramethyl-1-piperidinyloxy
Udh: Uronate dehydrogenase
UxaC: Uronate isomerase
Zwf: Glucose-6-phosphate dehydrogenase
